# The current state of artificial intelligence generative language models is more creative than humans on divergent thinking tasks

**DOI:** 10.1038/s41598-024-53303-w

**Published:** 2024-02-10

**Authors:** Kent F. Hubert, Kim N. Awa, Darya L. Zabelina

**Affiliations:** Department of Psychological Sciences, University of Arkansas, Fayetteville, AR 72701 USA

**Keywords:** Psychology, Human behaviour

## Abstract

The emergence of publicly accessible artificial intelligence (AI) large language models such as ChatGPT has given rise to global conversations on the implications of AI capabilities. Emergent research on AI has challenged the assumption that creative potential is a uniquely human trait thus, there seems to be a disconnect between human perception versus what AI is objectively capable of creating. Here, we aimed to assess the creative potential of humans in comparison to AI. In the present study, human participants (N = 151) and GPT-4 provided responses for the Alternative Uses Task, Consequences Task, and Divergent Associations Task. We found that AI was robustly more creative along each divergent thinking measurement in comparison to the human counterparts. Specifically, when controlling for fluency of responses, AI was more original and elaborate. The present findings suggest that the current state of AI language models demonstrate higher creative potential than human respondents.

## Introduction

The emergence of ChatGPT—a natural language processing (NLP) model developed by OpenAI^1^ to the general public has garnered global conversation on the utility of artificial intelligence (AI). OpenAI’s Generative Pretrained Transformer (GPT) is a type of machine learning that specializes in pattern recognition and prediction and has been further trained using Reinforcement Learning from Human Feedback (RLHF) so that ChatGPT responses would be indistinguishable from human responses. Recently, OpenAI^1^ has advertised the new model (GPT-4) as “more creative” particularly “on creative and technical writing tasks” in comparison to previous versions, although there are arguably semantic limitations such as nonsensical answers or the possibilities of incorrect information generation^2^. Given the accessibility of AI models in the current climate, research across a variety of domains has started to emerge, thus contributing to our growing understanding of the possibilities and potential limitations of AI.

Creativity as a phenomenological construct is not immune to the effects of AI. For example, researchers have begun to assess AI models to determine appropriate design solutions^3^ and logical reasoning^4^. These assessments focus on convergent thinking, i.e., determining one optimal solution to a pre-defined problem^5^. Traditionally, convergent thinking assumes an optimal single solution path and can be assessed through traditional intelligence measures or synthesis tasks. Although convergent thinking emphasizes single optimal solutions, this does not negate the potential for original or non-obvious solutions. However, convergent thinking tasks by design typically do not allow for flexible or out-of-the-box thinking. In contrast, divergent thinking involves generating multiple creative solutions to a problem which allows for the flexibility to determine multiple creative solutions^6^. Creativity researchers commonly focus on divergent creativity (in comparison to convergent creativity), given the associative mechanisms that allude to people’s ability to generate creative solutions (i.e., creative potential). Specifically, divergent thinking is considered an indicator of a person’s creative potential, but this does not guarantee creative achievement^7^. Instead, creative potential can be indicative on future capability, rather than an immediate trait that determines *if* someone is creative. Accordingly, a person’s creative potential has been captured via divergent thinking tasks such as the Alternative Uses Task [AUT^6,7^] or the Consequences Task [CT^8,9^]. Divergent thinking tasks can be evaluated along three dimensions: fluency (number of responses), originality (response novelty), and elaboration (length/detail of response). Responses in each category are given scores (i.e., according to each task) and used to assess individual differences in divergent creativity, or in other words, a person’s creative potential.

Given the emergence of OpenAI’s GPT-4 as a large language model, research has begun to empirically assess the creative potential of artificial intelligence language models through divergent thinking tasks. On one hand, some researchers argue that the human cognitive mechanisms present during creative tasks are not present in AI, and thus the creative potential of artificial intelligence can only reflect artificial creativity^10^. On the other hand, computational creativity suggests parallel networks that reflect the mechanisms of how humans go through iterative, deliberative, and generative creative processes which aid in the ability to determine creative solutions^11^. Although these aspects have been shown to aid in creative solutions, humans can experience idea fixedness, which can act as a roadblock to other creative solutions. Machines, however, will not experience this phenomenon in a metacognitive way due to computationally trained models that streamline a machine’s direct responses to a prompt^12–14^. Instead, a machine’s fixedness may perhaps reflect the training data of the model which could be argued is a computational consideration, rather than a creative one.

Furthermore, computational researchers have posed increasing debate on the creative capabilities of artificial intelligence models^15^ by asking questions such as: How are machines capable of determining what is creative? At present, AI’s inability to explicitly determine why or if something is creative is then compensated through human-assistance. For example, human intervention is necessary for inputting appropriate and relevant data to train the model and shape outputs to become more linguistically natural^16,17^. This computational limitation suggests that AI is not capable of divergent creativity due to the lack of metacognitive processes (i.e., evaluation, task motivation) because AI could not generate creative ideas or reiterate on existing ideas without the intervention (i.e., input) of a human user^10^. Similarly, emotions have been seen as an integral part of creativity such that emotions help dictate states of flow or mind-wandering that aid in creative processes^18^. However, AI may not necessarily need to rely on metacognitive or affective processes to generate novel ideas^19^ due to the computational framework. Thus, inner processes that contribute to human creativity may be a philosophical argument within artificial creativity models^20^.

As briefly reviewed, the creative capabilities of artificial intelligence, thus far, have scientifically and philosophically varied [e.g.,^10,20^]. Researchers posit humanistic and computational considerations of the creative potential of AI, however, the accessibility of tools to artificially generate products or ideas have given researchers the opportunity to evaluate public perception. For instance, people think more highly of generated artworks if they were told the artworks were created by humans but not AI^21,22^. The expectancy that AI generated products or ideas are less creative or hold less aesthetic value than human-created artworks appear to depend on implicit anti-AI biases^22–24^, as AI has been found to be indistinguishable from human-created products^25–27^. People’s inability to distinguish between human and AI-created products supports the feasibility of AI having creative potential.

Indeed, AI has been found to generate novel connections in music^28^, science^26^, medicine^29^, and visual art^30^ to name a few. In assessments of divergent thinking, humans outperformed AI on the Alternative Uses Task^31^, but it is noteworthy that the authors propose a possible rise in AI capabilities given future progress of large language models. In fact, recent studies have found that AI divergent creativity matched that of humans using a later version of GPT-4^32,33^. Researchers have continued to demonstrate that the current state of LLM’s frequently score within the top 1% of human responses on standard divergent thinking tasks such as the Alternative Uses Task^32–34^. Additional studies utilizing other divergent thinking tasks have also reported findings that paint a more nuanced picture. For example, when scores were compared between humans and GPT-4 on a Divergent Associations Task (DAT^35^), the researcher found that GPT-4 was more creative than human counterparts^36^. Recent research on OpenAI’s text-to-image platform DALL▪E has reported similar findings^37^ and suggests that OpenAI models could match or even outperform humans in combinational creativity tasks. Given the research on AI creativity thus far, OpenAI’s advertorial claims that GPT-4 is “more creative” may hold more merit than anticipated.

## Current research

Thus far, the novelty of OpenAI’s ChatGPT has posed more questions that have yet to be examined. Although creativity has considered to be a uniquely human trait^38^, the emergence of OpenAI’s generative models suggests a possible shift in how people may approach tasks that require “out of the box” thinking. Thus, the current research aims to examine how divergent creativity (i.e., fluency, originality, elaboration) may differ between humans and AI on verbal divergent thinking tasks. To our knowledge, this is one of the first studies to comprehensively examine the verbal responses across a battery of the most common divergent thinking tasks (i.e., Alternative Uses Task, Consequences Task, and Divergent Associations Task) with novel methodology by matching the fluency of ideas between human subjects and ChatGPT. We anticipate that AI may demonstrate higher creative potential in comparison to humans, though given the recency of AI-centered creativity research, our primary research questions serve as exploratory in nature.

## Methods

### Participants

#### Human participation

Human participants (N = 151) were recruited via Prolific online data collection platform in exchange for monetary compensation of $8.00. Participants were limited to having a reported approval rating above 97%, were proficient English speakers, and were born/resided in the USA. Average total response time for completing the survey was 34.66 min. A statistical sensitivity analysis indicated that we had sufficient power to detect small effects with the present sample size (*f*^2^ = 0.06, 1 − *β* = 0.80). The present study was performed in accordance with the Declaration of Helsinki and was approved by the Institutional Review Board for Human Subjects Research at the University of Arkansas. All participants provided informed consent prior to the start of the study. All statistical analyses were conducted in R studio^39^. See Table 1 for participant demographics.

**Table 1:** Demographics of human sample (N = 151).

	*M* (*SD*) or n (%)
Age	41.21 (12.18)
Gender	
Female	58 (38%)
Male	93 (62%)
Ethnicity	
White or European American	102 (68%)
Black or African American	21 (14%)
Asian or Asian American	11 (7.1%)
Hispanic or Latinx	7 (5%)
Multiracial	10 (7%)
Education	
Less than high school	3 (2%)
High school graduate	26 (17%)
Some college	28 (19%)
2 year degree	12 (8%)
4 year degree	62 (41%)
Professional degree	18 (12%)
Doctorate	2 (1%)

#### AI participation

Artificial participants were operationalized as ChatGPT’s instancing feature. Each ChatGPT session was considered an independent interaction between the user and GPT interface. Here, we prompted separate instances per creativity measure (as detailed below) which resulted in artificial participation sessions. For example, we used a single session instance to feed each prompt and aggregated each prompt response into a data file. In total, we collected 151 instances which represent AI’s participation for a balanced sample. For two of the creativity measures (Alternative Uses Task and Consequences Task), which are the only timed tasks, fluency was matched 1:1 such that the number of responses for both groups is equal on these timed tasks. Fluency scores of each human respondent were first calculated to match 1:1 for each GPT-4 instance for the Alternative Uses Task and Consequences Task (detailed below). Only valid responses were retained. For example, human participant #52 had a total fluency score of 6, thus GPT-4 instance #52 was instructed to provide 6 responses.

### Creativity measures

#### Alternative uses task

The Alternate Uses Task (AUT^6^) was used to test divergent thinking. In this task, participants were presented with a common object (‘fork’ and ‘rope’) and were asked to generate as many creative uses as possible for these objects. Responses were scored for fluency (i.e., number of responses), originality (i.e., uniqueness of responses), and elaboration (i.e., number of words per valid response). Participants were given 3 min to generate their responses for each item. Following prior research^40^, instructions for human respondents on the AUT were:

*For this task, you'll be asked to come up with as many original and creative uses for [item] as you can. The goal is to come up with creative ideas, which are ideas that strike people as clever, unusual, interesting, uncommon, humorous, innovative, or different.*

*Your ideas don't have to be practical or realistic; they can be silly or strange, even, so long as they are CREATIVE uses rather than ordinary uses.*

*You can enter as many ideas as you like. The task will take 3 minutes. You can type in as many ideas as you like until then, but creative quality is more important than quantity. It's better to have a few really good ideas than a lot of uncreative ones. List as many ORIGINAL and CREATIVE uses for a [item]*.

Because the goal was to control for fluency, we excluded prompt parameters such as 'quantity' from the GPT-4 instructions. Similarly, GPT does not need timing parameters in comparison to humans because we denoted the specific number of responses required. See below for instructions used per GPT instance:

*For this task, you'll be asked to come up with as original and creative uses for [item] as you can. The goal is to come up with creative ideas, which are ideas that strike people as clever, unusual, interesting, uncommon, humorous, innovative, or different.*

*Your ideas don't have to be practical or realistic; they can be silly or strange, even, so long as they are CREATIVE uses rather than ordinary uses. List [insert fluency number] ORIGINAL and CREATIVE uses for a [item].*

#### Consequences task

The Consequences Task (CT^8,9^) is part of the verbal section of the Torrance Test of Creative Thinking (TTCT) that provides prompts to hypothetical scenarios (i.e., what would happen if humans no longer needed to sleep?). Similar to the AUT, people respond to as many consequences to the prompt as they can within a given timeframe. Responses were scored for fluency (i.e., number of responses), originality (i.e., uniqueness of responses), and elaboration (i.e., number of words per valid response). General task instructions for human respondents were:

*In this task, a statement will appear on the screen. The statement might be something like "imagine gravity ceases to exist". For 3 minutes, try and think of any and all consequences that might result from the statement. Please be as creative as you like. The goal is to come up with creative ideas, which are ideas that strike people as clever, unusual, interesting, uncommon, humorous, innovative, or different.*

*Your responses will be scored based on originality and quality. Remember, it is important to try to keep thinking of responses and to type them in for the entire time for the prompt.*

*REMINDER: In this task, a statement will appear on the screen. The statement might be something like "imagine gravity ceases to exist". For 3 minutes, try and think of any and all consequences that might result from the statement. Do this as many times as you can in 3 min.*

*The screen will automatically change when the time is completed. Remember, it is important to try to keep thinking of responses and to type them in for the entire time for the prompt.*

Participants were given two prompts shown independently: “Imagine humans no longer needed sleep,” and “Imagine humans walked with their hands.” The two CT prompts have been extensively used in research on divergent thinking^41–43^. Similar to the AUT, fluency and timing parameters were excluded from the GPT instructions on the CT:

*In this task, a statement will appear on the screen. The statement might be something like "imagine gravity ceases to exist". Please be as creative as you like. The goal is to come up with creative ideas, which are ideas that strike people as clever, unusual, interesting, uncommon, humorous, innovative, or different. Your responses will be scored based on originality and quality.*

*Try and think of any and all consequences that might result from the statement. [Insert scenario]. What problems might this create? List [insert fluency number] CREATIVE consequences.*

#### Divergent associations task

The Divergent Association Task (DAT^35^) is a task of divergent and verbal semantic creative ability. This task asks participants to come up with 10 nouns as different from each other as possible. These nouns must not be proper nouns or any type of technical term. Pairwise comparisons of semantic distance between the 10 nouns are calculated using cosine distance. The average distance scores between all pairwise comparisons are then multiplied by 100 that results in a final DAT score (https://osf.io/bm5fd/). High scores indicate longer distances (i.e., words are not similar). Task instructions for both human participants and GPT-4 were:

*Please enter 10 words that are as different from each other as possible, in all meanings and uses of the words. The rules: Only single words in English. Only nouns (e.g., things, objects, concepts). No proper nouns (e.g., no specific people or places). No specialized vocabulary (e.g., no technical terms). Think of the words on your own (e.g., do not just look at objects in your surroundings).*

There were no time constraints for this task. The average human response time was 126.19 s (*SD* = 90.62) and the average DAT score was 76.95 (*SD* = 6.13). We scored all appropriate words that participants gave. Participants with fewer than 7 responses were excluded from data analysis (n = 2). Instructions were identical for the GPT-4 to the human instructions.

## Procedure

Human participants’ responses were collected online via Qualtrics. The entire study took on average 34 min (*SD* = 13.64). The order of the creativity tasks was counterbalanced. The online study used two attention checks randomly presented throughout the study. Each attention check allowed one additional attempt. Participants who failed two attention checks were removed from all analyses (N = 2). After providing their responses to each task, participants answered demographics questions.

GPT-4 procedural responses were generated through human-assistance facilitated by the first author, who provided each prompt in the following order: AUT, CT, and DAT. We did not have to account for typical human-centered confounds such as feelings of fatigue^44,45^ and order biases^44^ as these states are not relevant confounds in AI, thus the order of tasks was not counterbalanced.

### Research disclosure statement

All variables, measurements, and exclusions for this article’s target research question have been reported in the methods section.

## Results

### Creativity scoring

Both human and GPT-4 responses were cleaned to remove any instances that were incomplete or inappropriate at two stages: First, human responses that did not follow instructions from the task or were not understandable as a use (AUT; 0.96% removed) or a consequence (CT; 4.83%) were removed. Only valid human responses were used in matching for GPT fluency; Second, inappropriate or incomplete GPT responses for the AUT (< 0.001% removed) and CT (< 0.001% removed) were removed. Despite matching for fluency, only valid responses in both groups were used in subsequent analyses.

Traditional scoring methods of divergent thinking tasks have required human ratings of products or ideas and are assumed to be normative tasks (i.e., consensus will eventually be met with more raters). Here, we used the Open Creativity Scoring tool [OCS^46^] to automate scoring of semantic distance objectively by capturing the originality of ideas by assigning scores of the remoteness (uniqueness) of responses. Unlike human scoring which requires multiple factors of consideration (e.g., fatigue, biases, time, cost^47^) which could result in potential confounds, automated scoring tools such as OCS circumvent the human-centered issues and has been found to robustly correlate with human ratings^46^.

Open Creativity Scoring tool (OCS^46^) was used to score both the AUT and CT tasks. Specifically, the semantic distance scoring tool^17^ was used, which applies the GLoVe 840B text-mining model^48^ to assess originality of responses by representing a prompt and response as vectors in semantic space and calculates the cosine of the angle between the vectors. The OCS tool also scores for elaboration by using the stoplist method^46^. The prompts for the AUT were “rope” and “fork” and the prompts for the CT were “humans no sleep” and “humans walked hands.”

### Preliminary results

Descriptive statistics for all tasks are reported in Tables 2 and 3. Fluency descriptive statistics are reported in Table 2. Semantic distance descriptive statistics are reported in Table 3.

**Table 2:** Descriptive statistics of fluency for alternative uses task, consequences task, and divergent associations task responses for human and GPT-4 samples.

Prompt	*M* (*SD*)	Median	Skew	Kurtosis
Human				
Fork (AUT)	6.82 (3.67)	6	1.79	4.67
Rope (AUT)	7.06 (3.92)	6	1.07	1.17
No more sleep (CT)	5.98 (3.09)	5	1.45	3.48
Walk on hands (CT)	5.44 (3.30)	5	2.73	15.20
DAT	9.72 (0.62)	10	− 2.73	8.18
GPT-4				
Fork (AUT)	6.87 (3.66)	6	1.80	4.69
Rope (AUT)	7.13 (3.95)	6	1.03	1.01
No more sleep (CT)	5.72 (3.03)	5	1.39	3.28
Walk on hands (CT)	5.27 (3.26)	5	2.87	16.60
DAT	9.97 (0.18)	10	− 5.25	25.93

Skewness and kurtosis of DAT fluency was expected due to the task requiring 10 responses. Only valid and legible DAT responses were retained between both groups. *AUT* Alternative Uses Task, *CT* Consequences Task, *DAT* Divergent Associations Task.

**Table 3:** Descriptive statistics of originality using semantic distance for alternative uses task, consequences task, and divergent associations task responses for human and GPT-4 samples.

Prompt	*M* (*SD*)	Median	Skew	Kurtosis
Human				
Fork (AUT)	0.79 (0.04)	0.79	− 0.35	0.50
Rope (AUT)	0.68 (0.06)	0.68	0.03	0.03
No more sleep (CT)	0.67 (0.05)	0.67	0.18	− 0.28
Walk on hands (CT)	0.67 (0.06)	0.67	− 0.58	1.27
DAT	76.95 (6.13)	77.58	− 0.85	1.5
GPT-4				
Fork (AUT)	0.84 (0.02)	0.84	− 0.14	− 0.48
Rope (AUT)	0.79 (0.02)	0.80	− 0.59	1.00
No more sleep (CT)	0.71 (0.02)	0.71	0.05	0.34
Walk on hands (CT)	0.73 (0.01)	0.73	− 0.13	0.61
DAT	84.56 (3.05)	84.79	− 0.29	− 0.48

*AUT* Alternative Uses Task, *CT* Consequences Task, *DAT* Divergent Associations Task.

### Primary results

#### Alternative uses task

As expected, an independent sample *t*-test revealed no significant differences in total fluency due to controlling for fluency (as detailed above) between humans (*M* = 6.94, *SD* = 3.80) and GPT-4 (*M* = 7.01, *SD* = 3.81), *t*(602) = 0.21, 95% CI [− 0.54, 0.67], *p* = 0.83.

**Figure 1:**
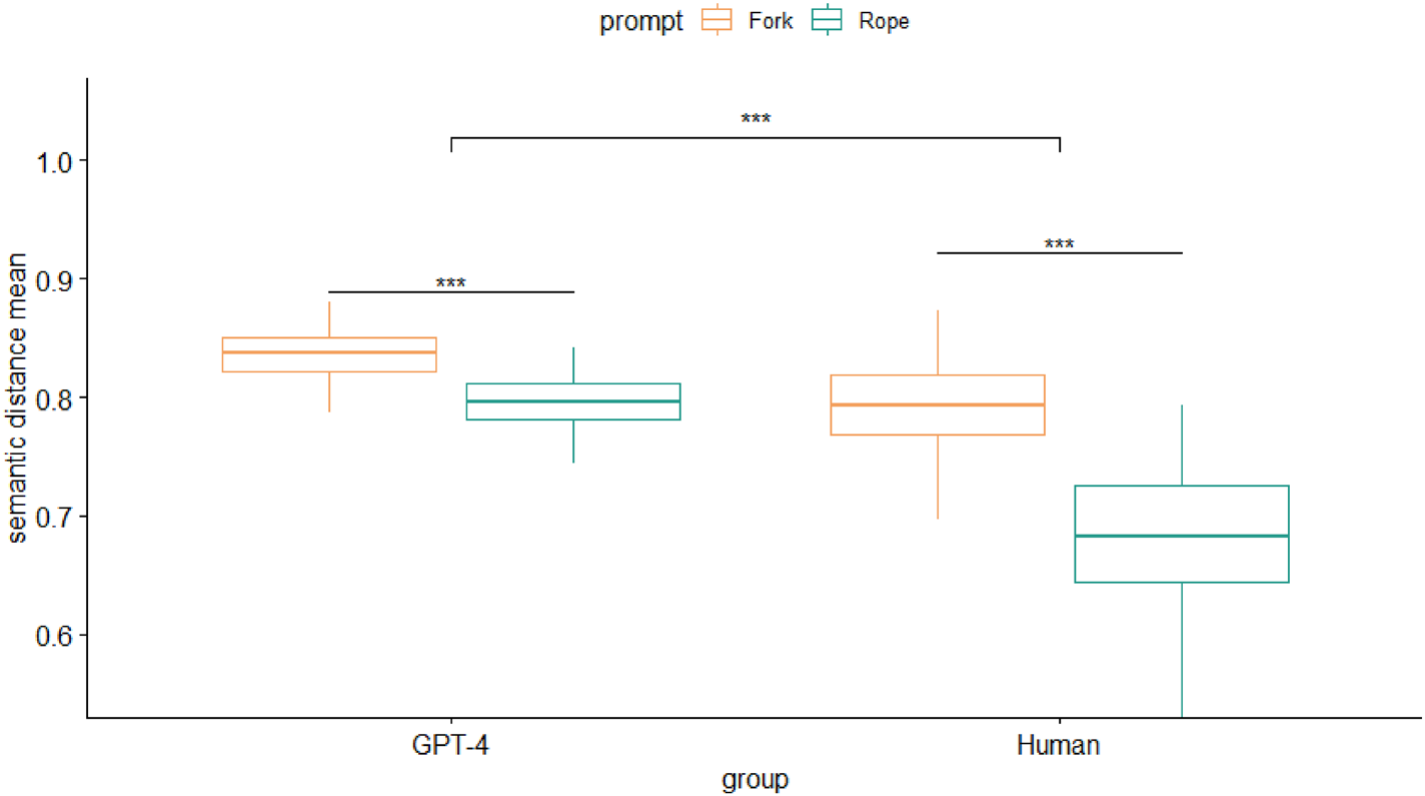
Analysis of variance of originality on the alternative uses task.

To assess originality of responses via semantic distance scores, we conducted a 2 (group: human, GPT-4) X 2 (prompt: ‘fork, rope) analysis of variance. The model revealed significant main effects of group (*F*(1, 600) = 622.10,* p* < 0.001, *η*^2^ = 0.51) and prompt (*F*(1, 600) = 584.50, *p* < 0.001, *η*^2^ = 0.49) on originality of responses. Additionally, there were significant interaction effects between group and prompt, *F*(1, 600) = 113.80, *p* < 0.001, *η*^2^ = 0.16. Particularly, both samples had higher originality scores for the prompt ‘fork’ in comparison to ‘rope,’ but GPT-4 scored higher in originality, regardless of prompt. Tukey’s HSD post hoc analysis showed that all pairwise comparisons were significantly different (*p* < 0.001) aside from the human ‘fork’ and GPT-4 ‘rope’ originality (*p* = 0.989). Overall, GPT-4 was more successful at coming up with divergent responses given the same number of opportunities to generate answers compared to the human counterpart and showed higher originality but only for specific prompts (Fig. 1).

Next, we compared elaboration scores between humans and GPT-4. Fluency scores differ from elaboration in the sense that fluency accounts for each coherent response whereas elaboration quantifies the number of words per valid response. For example, a person could respond “you could use a fork to knit or as a hair comb.” In this example, the fluency would be 2 (knitting instrument and comb), but the elaboration would be 12 (number of words used in the response). The results of an independent *t*-test revealed that elaboration was significantly higher for GPT-4 (*M* = 15.45, *SD* = 6.74) in comparison to humans (*M* = 3.38, *SD* = 2.91), *t*(602) = 28.57, 95% CI [11.24, 12.90], *p* < 0.001.

#### Consequences task

As expected, an independent *t*-test revealed no significant differences in total fluency between humans (*M* = 5.71, *SD* = 3.20) and GPT-4 (*M* = 5.50, *SD* = 3.15), *t*(621) = 0.82, 95% CI [− 0.29, 0.71], *p* = 0.41.

**Figure 2:**
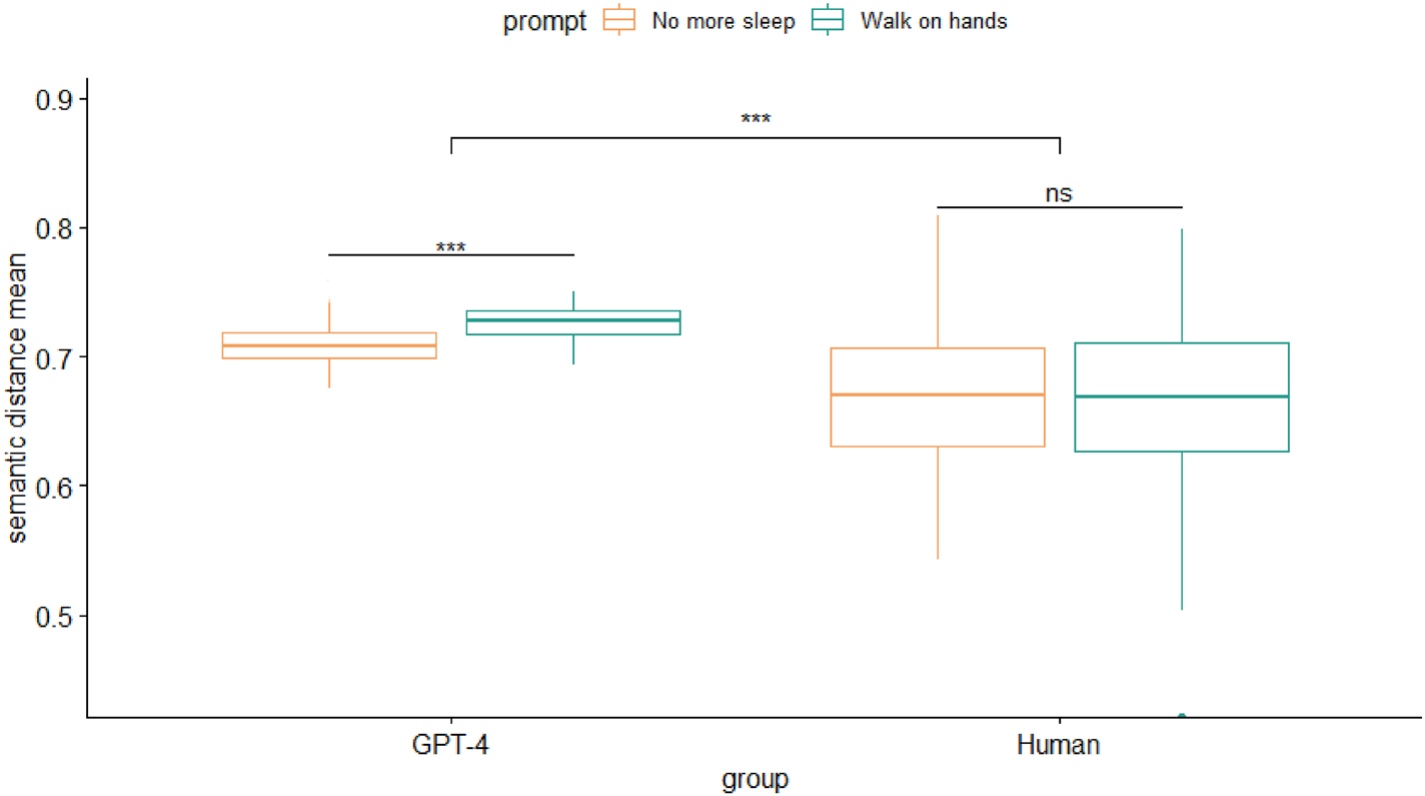
Analysis of variance of originality on the consequences task.

To assess originality of responses via semantic distance scores, we conducted a 2 (group: human, GPT) X 2 (prompt: ‘no more sleep,’ ‘walk on hands’) analysis of variance. The model revealed significant main effects of group (*F*(1, 619) = 622.10, *p* < 0.001, η^2^ = 0.51) and prompt (*F*(1, 619) = 584.50, *p* < 0.001, *η*^2^ = 0.49) on the originality of responses. Additionally, there were significant interaction effects between group and prompt, *F*(1, 619) = 113.80, *p* < 0.001, *η*^2^ = 0.16. Particularly, originality was marginally higher for the prompt ‘walk on hands’ in the GPT sample, although there were no significant differences in originality in the human sample between the two prompts. Tukey’s HSD post hoc analysis showed that all pairwise comparisons were significantly different (*p* < 0.001) aside from the human responses for both prompts (*p* = 0.607). Overall, GPT-4 was more successful at coming up with more divergent responses given the same number of opportunities compared to the human counterparts, and also showed higher originality dependent on prompt type (Fig. 2).

Next, we calculated the difference in elaboration between humans and GPT-4. The results of an independent I-test revealed that elaboration was significantly higher in the GPT-4 sample (*M* = 38.69, *SD* = 15.60) than in the human sample (*M* = 5.45, *SD* = 4.04), *t*(621) = − 36.04, 95% CI [− 35.04, − 31.45], *p* < 0.001.

### Divergent associations task

We assessed the qualitative aspect of the words generated in the DAT between both humans and GPT through word occurrence. Namely, the frequency of single-occurrence (non-repeating words within groups) and unique occurrence (words only occurring once between groups).

Humans had a higher number of single-occurrence words (n = 523) that accounted for 69.92% within the total group response in comparison to GPT’s number of single-occurrence words (n = 152) that accounted for 47.95% within the total group response (Table 4). In total, there was 9.11% (n = 97) of overlapping responses between both groups. Exclusively unique words that only occurred in the human responses accounted for 87.03% (n = 651) in comparison to unique GPT responses which accounted for 69.40% (n = 220).

**Table 4:** Top 20 most frequent words on the divergent association task in human and GPT-4 samples.

Human		GPT-4	
Word	Frequency	Word	Frequency
Dog	28	Elephant	98
Car	25	Symphony	55
Book	25	Microscope	51
Cloud	22	Quasar	44
Tree	21	Freedom	44
Computer	20	Dream	43
Water	16	Democracy	43
Chair	16	Love	40
Cat	16	Volcano	39
Moon	13	Quantum	39
Table	12	Philosophy	31
Sky	12	Microbe	27
Ocean	12	Galaxy	27
Mountain	12	Desert	26
Grass	12	Compass	22
Elephant	11	Microchip	19
Paper	10	Ocean	16
Flower	10	Justice	15
Fire	10	Harmony	15
Shoe	9	Dolphin	15

A chi-square test of independence was performed to examine the relationship between groups (GPT vs human) and word type (single occurrence vs unique occurrence). The relationship between these variables was not significant, $\chi$^2^ (1, *N* = 302) = 1.56, *p* = 0.211. This suggests that uniqueness and occurrences of words may not have necessarily aided either group in originality, but rather aided in word complexity.

Differences in semantic distance scores were calculated between human and GPT-4 DAT responses. An independent sample *t*-test revealed that GPT responses (*M* = 84.56, *SD* = 3.05) had higher semantic distances in comparison to human responses (*M* = 76.95, *SD* = 6.13), *t*(300) = 13.65, 95% CI [6.51, 8.71], *p* < 0.001. Despite human participants having a broader range of unique responses, the fluency uniqueness did not appear to advantage semantic distance scores when comparing groups.

## Discussion

The present study offers novel evidence on the current state of large language models (i.e., GPT-4) and the capabilities of divergent creative output in comparison to human participants. Overall, GPT-4 was more original and elaborate than humans on each of the divergent thinking tasks, even when controlling for fluency of responses. In other words, GPT-4 demonstrated higher creative potential across an entire battery of divergent thinking tasks (i.e., Alternative Uses Task, Consequences Task, and Divergent Associations Task).

Notably, no other study has comprehensively assessed multiple dimensions of the most frequently used divergent thinking tasks and AI. However, studies have begun to examine differences in divergent creativity between humans and AI, particularly after the public emergence of OpenAI’s ChatGPT, with findings showing that AI’s creative potential scores within the top 1% of human responses in terms of originality^32–34^. While there has been an influx in research examining the creativity of generative language models, to date only one previous study showed that humans outperformed GPT on the AUT (GPT-3^31^), while another study reported that later versions of GPT (GPT-4 showed similar, albeit slightly less, creative potential in comparison to humans^32^). Similarly, one previous study demonstrated that generative models were improved in GPT 4 compared to GPT 3.5, particularly in terms of fluency, but interestingly, not in terms of elaboration^49^ which suggests that the creative potential of these LLM’s are improving, particularly the ability to generate original ideas. Indeed, only one other study thus far has reported similar results that GPT outperformed humans on the DAT^36^, but the DAT is only one aspect of divergent thinking. Instead, the novelty of the present findings provides a foundation for future research to continue to examine multiple dimensions of divergent thinking and artificial intelligence.

While the present results suggest that the current state of AI models outperform humans on divergent thinking tasks by a significant margin, there are methodological considerations that could have contributed to the present results. To comprehensively examine creativity requires not only an assessment of originality, but also of the usefulness and appropriateness of an idea or product^50^. Traditionally, this has proven difficult to standardize in comparison to assessing originality given the multifaceted dimensions that contribute to assessments of appropriateness such as accounting for sociocultural and historical contexts. Semantic distance scores do not take into consideration the aforementioned variables; instead, the scores reflect the relative distance between seemingly related (or unrelated) ideas. In this instance, GPT-4’s answers yielded higher originality than human counterparts, but the feasibility or appropriateness of an idea could be vastly inferior to that of humans. Thus, we need to consider that the results reflect only a single aspect of divergent thinking, rather than a generalization that AI is indeed more creative across the board. Future research on AI and creativity needs to not only account for the traditional measurements of creativity (i.e., fluency, elaboration, originality) but also for the usefulness and appropriateness of the ideas.

Interestingly, GPT-4 used a higher frequency of repeated words in comparison to human respondents. Although the breadth of vocabulary used by human responses was much more flexible, this did not necessarily result in higher semantic distance scores. Flexibility, or number of categories of responses, has also been found to be smaller (i.e., more similar categories of words were generated) for AI in comparison to humans^34^. In other words, like our present results, humans came up with a wider range of responses, however, this did not indicate increased originality. These findings highlight the consideration that flexible thinking may be the strong point in human-centered divergent thinking.

More so, the complexity of words chosen by AI, albeit more concentrated in occurrence, could have more robustly contributed to the originality effects. For example, only AI used words that are non-tangible items (i.e., freedom, philosophy) whereas humans may have experienced a fixedness on generating ideas that are appropriate and observable. The differences between generated lists (incorporating tangible and non-tangible word) could inflate originality to be biased toward AI.

Similarly, we need to critically consider the uniqueness of words generated in DAT responses. There was a marginal overlap of responses between the human and the AI samples (9.11%), but humans responded with a higher number of single-occurrence words. Despite these differences, AI still had a higher semantic distance score. Prior research shows that in human respondent’s originality increases over time^51^. This increase is seen as an expansion of activation in an individual’s semantic network, which leads to more original responses^52^. Human responses on these DT tasks tend to follow a diminishing returns curve before reaching a plateau for an individual’s more original responses^53^. The higher levels of elaboration and semantic distance in AI responses suggests that the LLM processing possibly does not need this ramp-up time as seen in human responses, therefore LLM’s can respond with their highest level of original responses when prompted. Whereas humans may fixate on more obvious responses at first, this algorithmic trait could then serve as an aid in overcoming ideation fixedness in humans.

It is important to note that the measures used in this study are all measures of creative potential, but involvement in creative activities or achievements is another aspect of measuring a person’s creativity. Creative potential is not a guarantee for creative achievement; instead, we need to consider creative potential as an indicator of a person’s creative capabilities^7^. Here, AI was more original thus indicating higher creative potential, but this metric may more appropriately reflect the advancement of the algorithms these models were trained on in conjunction with human input. In other words, AI, unlike humans, does not have agency, thus AI creative potentials are dependent on the assistance of a human user to elicit responses. Therefore, the creative potential of AI is in a constant state of stagnation unless prompted.

Moreover, researchers have examined the interplay between creative potential and real-world creative achievements^54,55^ but this approach assumes human level creativity and is not able to account for artificial intelligence. AI can generate creative ideas, but it cannot be assumed that this potential would translate to achievement. The creative potential of AI is limited by the (lack of) autonomy of what the algorithms can create (i.e., creative potential) without the intervention of human assistance. Thus, future research should consider the conceptual implications of current measurements of creativity as implicated in applications in real-world settings and how generalizability at the intersection of potential and achievement may be a human-centric consideration.

The prevalence and accessibility of the internet has drastically shaped the way in which humans interact with language processing systems and search engines. LLM’s such as GPT-4 are now not an exception in ubiquity. Searching for information has multiple channels which were not previously available, and with these functions come an array of strategies to best find the desired information. Research has shown that younger people are better and more efficient in their search strategies online to find the information they want^56^, which suggests that exposure to search platforms acts as a practice in efficiency. Similar to interactions with GPT-4 and other AI platforms, humans may gradually navigate how to best utilize LLM’s. For information seeking tools like GPT-4, the creative potential has shown clear progression in capabilities, albeit there are still limitations such as response appropriateness and AI’s ability to generate idiosyncratic associations. Generative AI has demonstrated robustness in creative potential but has also shown weaknesses (i.e., less flexible thinking) that could then be supplemented by human assistance. Moving forward, future possibilities of AI acting as a tool of inspiration, as an aid in a person’s creative process, or to overcome fixedness is promising.

## Data Availability

All data associated with the present study is available at https://osf.io/xv6kh/.
